# Leg Length Discrepancy: Dynamic Balance Response during Gait

**DOI:** 10.1155/2018/7815451

**Published:** 2018-06-10

**Authors:** Nurul Azira Azizan, Khairul Salleh Basaruddin, Ahmad Faizal Salleh, Abdul Razak Sulaiman, Muhamad Juhairi Aziz Safar, Wan Mohd Radzi Rusli

**Affiliations:** ^1^School of Mechatronic Engineering, Universiti Malaysia Perlis, Pauh Putra Campus, 02600 Arau, Perlis, Malaysia; ^2^Department of Orthopaedics, School of Medical Science, Universiti Sains Malaysia, 16150 Kubang Kerian, Kelantan, Malaysia

## Abstract

Balance in the human body's movement is generally associated with different synergistic pathologies. The trunk is supported by one's leg most of the time when walking. A person with poor balance may face limitation when performing their physical activities on a daily basis, and they may be more prone to having risk of fall. The ground reaction forces (GRFs), centre of pressure (COP), and centre of mass (COM) in quite standing posture were often measured for the evaluation of balance. Currently, there is still no experimental evidence or study on leg length discrepancy (LLD) during walking. Analysis of the stability parameters is more representative of the functional activity undergone by the person who has a LLD. Therefore, this study hopes to shed new light on the effects of LLD on the dynamic stability associated with VGRF, COP, and COM during walking. Eighteen healthy subjects were selected among the university population with normal BMIs. Each subject was asked to walk with 1.0 to 2.0 ms^−1^ of walking speed for three to five trials each. Insoles of 0.5 cm thickness were added, and the thickness of the insoles was subsequently raised until 4 cm and placed under the right foot as we simulated LLD. The captured data obtained from a force plate and motion analysis present Peak VGRF (single-leg stance) and WD (double-leg stance) that showed more forces exerted on the short leg rather than long leg. Obviously, changes occurred on the displacement of COM trajectories in the ML and vertical directions as LLD increased at the whole gait cycle. Displacement of COP trajectories demonstrated that more distribution was on the short leg rather than on the long leg. The root mean square (RMS) of COP-COM distance showed, obviously, changes only in ML direction with the value at 3 cm and 3.5 cm. The cutoff value via receiver operating characteristic (ROC) indicates the significant differences starting at the level 2.5 cm up to 4 cm in long and short legs for both AP and ML directions. The present study performed included all the proposed parameters on the effect of dynamic stability on LLD during walking and thus helps to determine and evaluate the balance pattern.

## 1. Introduction

In human movement, stability plays an important role to help avoid sustaining serious injuries such as fall for those who are particularly unstable, and falls are likely to cause bone fractures in the long term. Leg length discrepancy (LLD), known as anisomelia [1], is a condition described when both legs are noticeably unequal in the lower extremities. A report from 2007 indicated that LLD is identified in almost 40% to 70% of the populations [2, 3]. Patients with an asymmetrical leg length will have a provoked postural control and minimal stability during standing and walking [4]. It is often developed by the changes in kinematics of the lower limb which involves altering the plantar flexion of the ankle on the short side with pelvic tilt whilst flexing the hip and knee on the long side. Low back pain and scoliosis are significant global health issues affecting those with LLD. The aetiology of LLD can be classified into two types—structural LLD, defined as the shortening of the bone structures, and functional LLD, defined as any mechanical changes that alter the posture of the lower extremities such as knee flexion/extension. In addition, the insole materials are comprehensively used to mimic LLD such as hard cork lift [5], flexible polyurethane [6], polypropylene [7], wooden boards [8], plywood [9], high-density ethylene vinyl acetate [10], sole of pelite [11], leather nylon mesh tissue, plastazote EVA and poron, thermoplastic alloy (TPA), and foot mask. A study conducted by Zhang et al. [7] points out that the changes of the foot placement and the restriction of ankle movements may be related to the sole materials/design and/or elevated height of pedestrians tested. Several attempts made by previous authors revealed that there were changes in gait for LLD greater than 2 cm to 3 cm [12]. For LLD greater than 2 to 3 cm [12], results show increased ground reaction force (GRF) and obvious changes in lower extremity kinetics and kinematics [13, 14]. Maeda et al. [5] measured body posture and dental occlusion during static standing with artificial LLD (0.1 cm, greater or less than 0.4 cm and 0.6 cm). They observed that weight distribution (WD) and the centre of pressure (COP) showed great significant difference between artificial LLD and control group (no LLD). In addition, several researches [1, 15, 16] noted that short leg exerted more forces than the long leg during static standing. Recently, review literature showed published findings of the effect of vertical ground reaction force (VGRF), COP, and COM as parameters to interpret stability in different cases, for example, cerebral palsy, amputees, and stroke patients [1]. Hsue et al. [17] proposed a study of the spatial relationship between COP and COM trajectories as well as interaction of COP-COM distance in order to quantify the dynamic balance of the children with cerebral palsy. It can be hypothesized that increasing the VGRF during single-leg support tends to produce uneven weight distribution in double-leg support. Both parameters will influence stability in the short leg when LLD is simulated. The greater peak-to-peak COP-COM separation leads to provide a greater requirement of postural control deficiency. Postural control increases the moment arm so that the ground reaction force could act for momentum generation. This also may increase moment arm for the body weight vector acting around the centre of the joint rotation. Further, the increase in LLD height will lead to higher value of RMS COP-COM distance and provoke instability. It can characterize the leg without LLD (normal) and leg with LLD and thus assist to compare and differentiate balance patterns. Hence, understanding of these parameters that help with stability in gait biomechanics is essential to help individuals suffering from this pathology that could affect their daily activities, particularly walking. Throughout this study, the subject needs to acclimate to the imposed discrepancy after the insoles thickness has been applied. To the best of our knowledge, no previous study has assessed the occurrence of body postural stability in LLD during dynamic walking. The present study was therefore designed to assess the effect of LLD on the dynamic balance stability with variation of LLD severity.

## 2. Method

### 2.1. Subject

Data were collected from a group of 18 male subjects who were chosen from the university population without any self-reported LLD or any lower limb abnormal pathology. Average age was 22.4 ± 1.9 years, with an average overall height of 166.9 ± 5.3 cm, overall weight of 62.9 ± 7.8 kg, and a BMI of 22.5 ± 1.7 kg/m^2^. Subjects were advised on the purpose of the study and the protocol prior to the experiment. Subject assent and written consent form for each subject and protocol of the study was approved by the local ethics committee.

### 2.2. Procedure and Data Collections

The subjects wore proper sports attire and a modified Helen Hayes marker set of 30 passive markers attached on anatomical landmarks such as the trunk, pelvis, shank, thigh, and foot (heel, 1st, 3rd, and 5th metatarsal) segments for both legs with the anthropometry data. This weighted sum of segmental model was sufficient to compute the whole-body COM. Passive markers were attached over the palpable anatomical landmark which was relevant to facilitate calculation of the total COM positions and velocity [18]. Tracking markers were secured at convenient locations to help tracking the centre of mass of the segment's body landmarks as suggested by Cappozzo et al. [19]. Marker position was calibrated using a two-second standing trial in a natural upright posture with their arms alongside their trunk. They were instructed to stand as still as possible with their body weight distributed evenly between their legs to capture static posture. Nine pieces of insoles each with 0.5 cm thickness were inserted consequently under the right foot as an induced LLD while the left represented the short leg. In this study, right leg was used as a dominant leg for all subjects during the experiment, and the added insoles were expected to cause an asymmetric gait pattern. Thereafter, the subject walked for about 1 min to adapt to the insoles and sandals provided until he felt comfortable to walk in a set of experiments. The experiment was replicated by the subjects until 4 cm of induced LLD and just a few minutes to rest before undergoing the next experiment to avoid any abstracted psychological effect in the adaptation of the asymmetrical gait.

**Figure 1:**
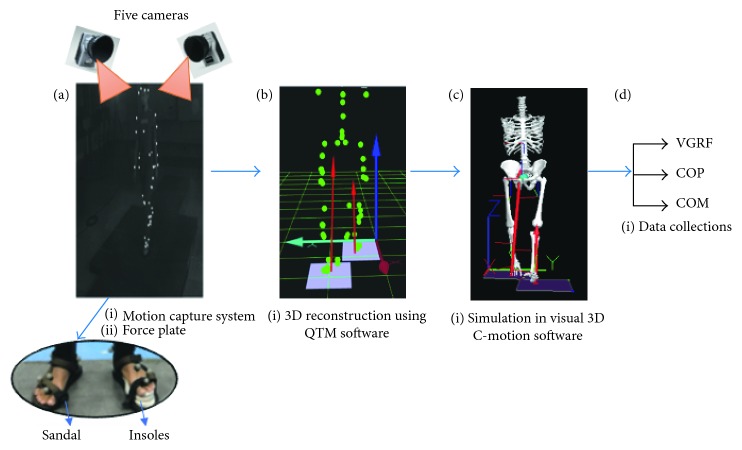
Overview of the experiment for walking with LLD. (a) Captured motion analysis and visualized position of reflective marker at any given time on the force plate. (b) Labelling all the marker trajectories and recording the subject's ground reaction force. (c) Simulation of the three-dimensional segmental skeleton. (d) Collecting the required data.

The experiment was carried out by initiating gait for each foot separately using two force plate Bertec corporation forces with a dimension of 60 cm × 40 cm × 10 cm at 200 Hz. Prior to the experiment, data were retrieved after a successful completion of walking with a minimum three to five trials of walk on a 7 m track lab and force plate. The walking speed was controlled for about 1.0 to 2.0 ms^−1^. Since only two forces plates were used to examine the GRFs and COP, the subject walked to start with foot initial contact with the long leg and ended with the foot contact on the same leg. Five Oqus motion analysis cameras and Qualysis Track Manager (QTM) software were used to record the three-dimensional kinematic data and segmental motions at 120 Hz. All the markers were labelled and fill gap marker trajectories were interpolated when necessary in the QTM software. Subsequently, data were exported to the biomechanics analysis processing C-Motion's Visual 3D motion analysis software version 3.91.0 to help simulate the segmental model. The GRFs, COP, and COM were computed, and their motion artefact was filtered by using a fourth-order Butterworth low-pass filter at 6 Hz for each model. The overview of the whole experiment is shown in Figure 1.

### 2.3. Data Analysis

Briefly, the GRF readings were recorded from each leg in a single-leg stance and a double-leg stance. In order to evaluate the body stability, the weight distribution (WD) and the vertical ground reaction force (VGRF) were computed according to the vertical direction of the arrow from the force plate exerted on the body during foot contact. In this study, single-leg stance was defined when the VGRF reached to the maximum value for each leg, whereas the double-leg stance was referred to as the maximum of total VGRF for both legs. VGRF values were normalized by each of the participant's body weight. The human COM motion moves in sinusoidal path from the inverted pendulum single-leg stance and double-leg stance, respectively. The COP is the sum of all ground forces and moment acting from the surface of foot placement during the gait cycle that was measured by the force plates. The COP and COM trajectories were computed from peak-to-peak maximal amplitudes in anterior-posterior (AP) and medial-lateral (ML) directions. The vertical direction was computed in COM trajectory only. Both trajectories were computed in a full gait cycle. The COP and COM trajectories were traced when foot contacted to the ground in two conditions, which are (1) without LLD (for 0 cm different) and (2) with LLD (for 0.5 cm to 4 cm different). The displacement of COP and COM trajectories was adopted based on the study of Hsue et al. [17] to evaluate the influence of the topographic involvement of this relationship to determine whether the COP and COM parameters could be used to discriminate between the levels of LLD and the control (no LLD). This also could identify how COP and COM parameters could help to define the factors that contribute to the LLD patients in imbalance gait disorder. The COP and COM data were normalized as dimensionless by each participant's leg length (LL) to eliminate the influence of individuals' stature [17]. Thus, the average root mean square (RMS) of COP-COM distance is used to identify the imbalance stability during gait. All the data were exported and analyzed further in MATLAB version R2017a (The MathWorks, Natick, MA, USA). The statistical result of RMS COP-COM distance was computed by using one-way ANOVA at the level of significance *p* < 0.05. Least significant difference (LSD) post hoc was used due to the small minimal difference value and multiple comparison of significance across every LLD's level. The optimal cutoff value through receiver operating curve (ROC) was used to identify the magnitude of inequality that can become pathological, which was adopted from Kumar and Indrayan [20]. The calculation of cutoff based on Youden index could compute the maximum sum of sensitivity and specificity with option empirical ROC graph. All data were analyzed using the statistical package for the social sciences (SPSS) software version 23 (IBM SPSS statistic, Inc.).

## 3. Results

### 3.1. VGRF in SLS and DLS

**Figure 2:**
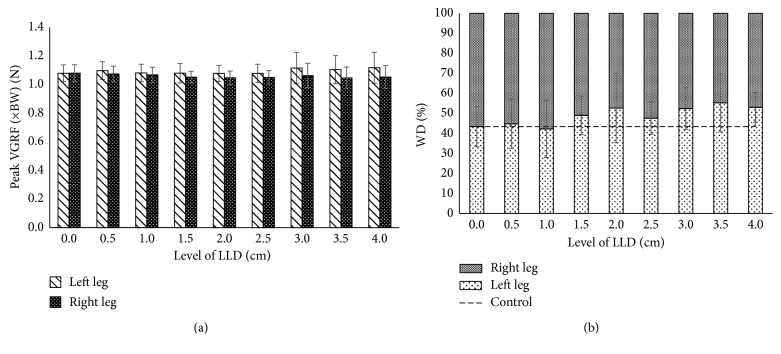
VGRF during the whole gait cycle. (a) Peak VGRF in single-leg stance. (b) WD in double-leg stance.

The first set of analyses examined the impact of VGRF in SLS and weight distribution during DLS. In order to access both of these parameters, all the data have been computed in mean and standard deviation as shown in Figure 2. In Figure 2(a), the graph clearly demonstrated that the value of peak VGRF was almost constant between the short leg and the long leg during single-leg stance. No obvious changes were noted between them. However, with successive increases in the level of LLD, the VGRF impact showed it moved towards the left leg which is represented as the short leg. However, the right leg (represented as the long leg) shows decreases due to the VGRF less distributed to the long leg during single-leg stance. The dotted line represents the control value from normal walking. During double-leg stance, the percentage of WD increased substantially in the short leg rather than the long leg. Besides that, it is clearly noticeable that the graph pattern started to change at 1.5 cm up to 4 cm, which shows significant difference between both legs when compared to no LLD (0 cm). A visible change in the graph was noted when induced LLD was at 2 cm and 3.5 cm because WD was shifted significantly into the short leg rather than the long leg consequently. At the glance, overall result shows weight distribution on the short leg rather than long leg during gait.

### 3.2. COM in AP, ML, and Vertical Directions

**Figure 3:**
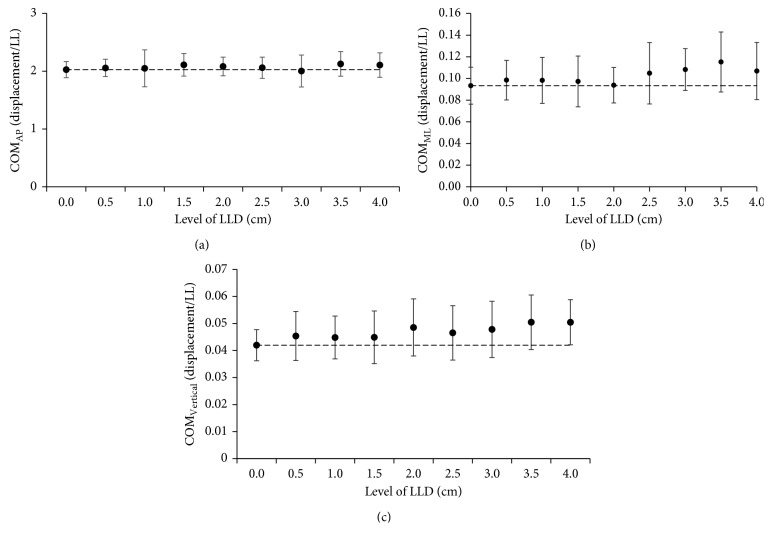
Effect of LLD levels on COM (during the whole gait cycle). Peak-to-peak displacement of COM trajectories in (a) AP direction, (b) ML direction, and (c) vertical direction.

The results of COM trajectories corresponding to the increments in LLD level are presented in Figure 3. The graphs present the result of mean and standard deviation values of peak-to-peak COM displacement in AP, ML, and vertical direction along the whole gait cycle. From the graph, the error bar is presented as standard deviation values and dotted plot shows mean values. Trajectories of COM in AP were almost constant with no obvious difference between no LLD (0 cm) and other increasing levels as shown in Figure 3(a). In average, COM in ML direction being increased unevenly through the whole gait cycle is presented in Figure 3(b). When the LLD level started increasing from 2.5 cm until 4 cm, there were obvious differences compared to no LLD. It has slight difference occurring at 0.5 cm up to 1.5 cm levels.  Figure 3(c) shows mean and standard deviation of peak-to-peak displacement across LLD level in vertical direction. Similarly, the result also presented changes at the level from 0.5 cm until 4 cm compared to no LLD. The obvious difference was found starting at level 2 cm.

### 3.3. RMS COP-COM Distance in AP and ML Directions

**Figure 4:**
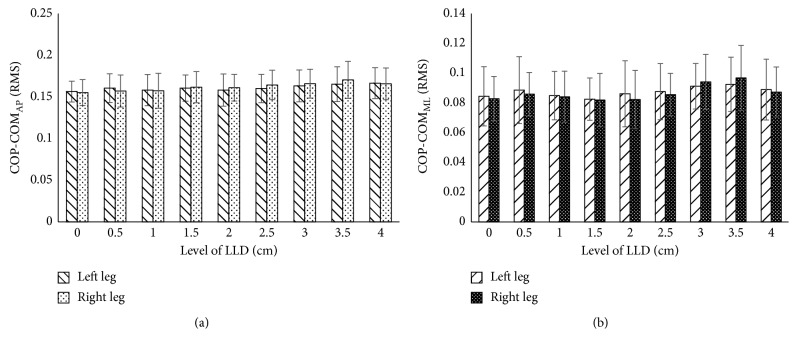
RMS of COP-COM distance in the whole gait cycles: (a) AP direction and (b) ML direction.

Figure 4 shows the result of mean and standard deviation RMS of COP-COM distance in AP and ML directions throughout the entire gait cycle. Interestingly, the graph shows direct correlation of the values of the average RMS between right leg (long leg) and left leg (short leg) from 0.5 cm up to 4 cm LLD level.  Figure 4(a) shows that the experiment data are almost constant for both legs when the subject walked in linear progression (AP direction). Slight differences occurred in the long leg rather than in the short leg. The data increased when LLD reached the level of 3.5 cm. Meanwhile, the RMS of COP-COM values remained stable at 0.5 cm, 1 cm, and 2 cm. However, at 4 cm LLD, there was no obvious change between both legs. In ML direction, the pattern is very different for RMS for the COP-COM distance as shown in Figure 4(b). Once the LLD level has been added, the obvious changes occurred at the level 3 cm and 3.5 cm of LLD , respectively. From this result, the RMS of COP-COM distance demonstrated in the short leg is substantially greater when compared to the long leg. Overall, strong evidence was found on the effect of RMS for COP-COM displacement in AP and ML directions on LLD during gait.

## 4. Discussion

It is concurred that the understanding and knowledge of how VGRF, COM, and COP trajectories as a mechanism of dynamic stability is required during walking. It is possible to distinguish the determinants of stability in asymmetry gait by visual inspection for severe LLD; however, it is hard to prove and difficult to quantify the difference appropriately for mild levels of LLD for the human eye. Therefore, this study set out to assess the effect of VGRF, COM, and COP trajectories, as well as RMS difference between each COP-COM distance parameters on the dynamic stability of LLD during walking.

The results from this study indicates that the typical performance and appearance of the VGRF during SLS and DLS. Our finding on the effect of Peak VGRF during single-leg stance and WD during double-leg stance demonstrates that short leg tends to receive more forces compared to the long leg. This present study findings are in tandem and reinforced with the previously published research [4, 8, 21, 22]. In contrast to dynamic walking, the uneven distribution on the short leg can be partially counteracted by extending the contralateral knee during static standing. This justification is reinforced in a study by Kim et al. [21] that various balance disorders could be investigated on a single-leg test to quantify the postural steadiness quantitatively. These results are in line with previous findings which revealed that individuals with LLD will alter at the spine, pelvis, and in the lower extremity joints when the short leg sustained a greater proportion of loading rate [23]. In a study of children with spastic hemiplegia conducted by Eek et al.[24], it was shown that postural stability may change due to the abnormalities with more flexion in the uninvolved leg, short leg compared to the long leg. However, pelvic and/or spinal joints may be distracted and cause a worse problem from this posture as justified by Walsh et al. [11] who revealed that pelvic obliquity is the most common way to compensate leg length discrepancy in the experimental study tested by different heights of shoe rises.

During active phases of the gait cycle (dynamic walking), the weight distribution shifted on the short leg logically may induce perturbation in postural control and stability. According to Bonnet et al. [4], the biggest challenge during the double-leg stance is to regain stability and the control of the foot placement due to a perturbation of the hip load/unload mechanism which induces more horizontal acceleration interaction with the COM [4]. This observation is strengthened by previous studies [3, 6, 18], which clarified that the greater loading on the short leg may be explained by the fact that step-down distance is greater in the short leg when compared to the long leg in the transition of the long leg to the short leg in stance phase. Owing to this, the forces would be higher as transferring of the weight is from a greater vertical height. This observed increase in weight acceptance force could be ascribed to the shorter time in peak force with concomitant changes in joint kinematics [25, 26]. These disproportionate forces and greater loading at the short leg could have affected soft tissue damage [27]. Nevertheless, our results showed that the long leg had a lower weight distribution compared to no LLD (0 cm). In contrary, Aiona et al. [28, 29] found that the majority of the total work was subjected to the long leg. Predominantly, more work influence and emerge at the location of the discrepancy which is associated with the increased force at the ankle [30], hip on the short leg and total force on the long leg. In addition, increasing of stiffness at the ankle joint [28] could enhance the output structure of the ground reaction force that lead to the better balance performance during double-leg stance. This assumption was supported by Zhang et al. [7] who found that the range of motion contributes to the severity effect on the ankle, hip, and knee during gait.

On the other hand, it is difficult to explain the configuration on the mechanism of dynamic stability results, but it might be related to the delineation “walking by falling” poor postural control through LLD during gait. COP, COM, and interaction of COP-COM distance can provide a very persuasive arguments and information about the perturbation in asymmetry gait. In the present study, COM in AP direction showed no obvious changes because COM moves constantly in forward progression beyond the base of support during walking. However, ML and vertical direction show COM shifted far away from control (0 cm) as LLD level increased. Relatively, the supporting foot and the position of COM demonstrated that the body was in a continual state of dynamic imbalance throughout the transition in single-leg to double-leg stance phase. As LLD level increased during gait, the larger lateral and vertical COM displacement indicates that the subject may not be able to generate enough hip abductor/adductor torque to keep the pelvis and trunk dropping to the side of the swing leg as discussed in a previous dynamic and stability study [17]. Therefore, passive lateral acceleration momentum is applied due to less muscular effort to lift one-foot step forward. Accordingly, the increase of VGRF in the short leg represents a greater displacement of COP trajectories on the short leg throughout the whole gait cycle. Due to this, when the subject walked with the increment insoles, the displacement of COM in ML and vertical directions was distracted within the base of support. Assogba et al. [3] and Kaufman and Miller [31] revealed that the displacement of COM during movement could be minimized when the subject walked with the compensatory mechanisms, and consequently, this shortens the long limb's time movement. Thereupon, it will reduce the energy consumption. Hsue et al. [17] proposed the simultaneous measurement of COP-COM relationship which can provide a more thorough and fruitful description of postural stability in gait since the COM and COP are crucial mechanisms during walking. As comparing RMS of COP-COM distance in AP direction, significant changes in the graph pattern with different values only exist in ML direction in the whole gait cycle.

**Figure 5:**
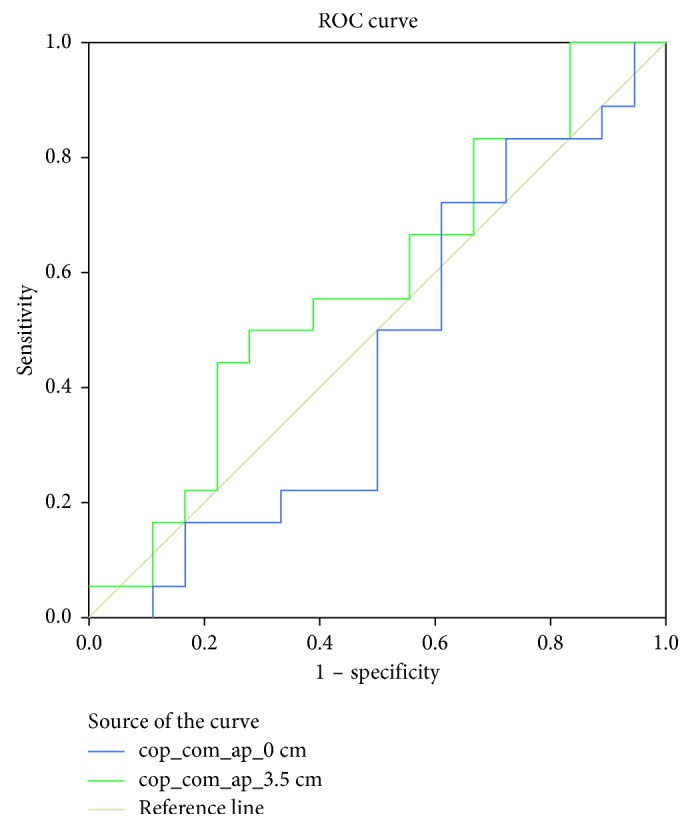
The example illustration of receiver operating curve for data in Table 1.

RMS of COP-COM distance in AP direction may not be accurate to contradistinguish upright stability between long leg and short leg. In the description of balance control, the COM motion relative to the COP [18, 32] was associated with the theory of the inverted pendulum. From this point of view, the subject shows increase in the sagittal plane (ML direction). On the other hand, in the present study, the result shows RMS of COP-COM distance occupied by long leg as an increment of insole at 3 cm and 3.5 cm. In contrast, this result shows disagreement with the findings of Hsue et al. [17], and Massaad et al. [33] showed that topographical types do not affect COM displacement. The result of RMS COP-COM distance in Figure 4 was supported with the statistic result by using one-way ANOVA with LSD post hoc test at the significance *p* < 0.05. There are significant differences occurring starting at the level 3 cm for long leg in AP and ML directions, whereas only LLD with 4 cm has significant difference in short leg for both directions. Since the RMS of COP-COM distance is still questionable in terms of providing the persuasive and informative data for LLD's patient, therefore, the cutoff value via ROC is useful in order to discriminate the normal and abnormal subject's stability during walking. The test with higher cutoff point would be considered affected. The sample of ROC in the present study is shown in Figure 5. In Table 1, the optimal cutoff points indicate that there are significant differences from level 2.5 cm up to 4 cm compared to normal walking (0 cm) for both legs' condition in AP and ML directions. At the glance, these cutoff values [34] are useful to support the result in Figure 4 in order to determine which inequality level becomes more pathological and allows the researchers to compare the performance of normal and abnormal tests. The LLD level from 0.5 cm to 2.0 cm indicates that cutoff points are less than normal walking (0 cm). Hence, the results suggest that this test is not sensitive to the small height of LLD.

The present study was the first to quantify the effect of LLD on the VGRF (single-leg stance) and WD (double-leg stance), COM and COP trajectories, and RMS of COP-COM distance that led to examination of the dynamic balance and postural stability during gait. However, further investigation might be necessary with the current approach of small variation of LLD level of balance impairments in the range of motion at ankle, knee, and hip. These variables would influence the dynamic balance in LLD throughout successive gait cycles. On the other hand, the new understanding in this current study should help to improve the biomechanical study on the impact of balance control during gait for LLD. This new understanding is necessary to establish enhancement of knowledge in balance control strategies in order to utilize clinched alongside fall counteractive action. Also, this information is needed especially to clinicians who, particularly throughout rehabilitation programmes, will be able to propose their patients to exchange a specific sum of body weight onto another side of leg during walking. Thus, overall information would be expected of improving those recuperating procedure for the treatment in maintaining the stability.

## 5. Conclusion

This study set out to explore the influence of LLD on VGRF (single-leg stance) and WD (double-leg stance), COM and COP trajectories, and RMS of COP-COM distance during gait. The result showed greater loading forces on the short leg as compared to the long leg. The effect of walking with LLD levels on the control of the body's COM motion relative to the COP was determined as the changes in the interaction between RMS of COP-COM distances. Also, there is significant increase in instability of body posture as the height of LLD increases. The finding in this study helps to increase our understanding of the mechanism of dynamic stability that is associated with LLD during walking. Although the study has successfully demonstrated the objectives outlined, it has certain limitations in terms of the interpretation of interaction between COP-COM distances; therefore, continued efforts are needed in order to make this variable more accessible to quantify the dynamic balance and postural stability with LLD during walking.

## Figures and Tables

**Table 1:** The value of optimal cutoff through ROC curve for RMS COP-COM distance.

*(a) Cutoff at the long leg in AP direction*									
Normal gait/LLD (cm)									
Variables	0.0	0.5	1.0	1.5	2.0	2.5	3.0	3.5	4.0
Sensitivity	0.389	0.663	0.112	0.774	0.445	0.282	0.332	0.334	0.332
1-specificity	0.276	0.668	0.054	0.495	0.276	0.168	0.163	0.221	0.165
Cutoff	0.161	0.148	0.146	0.150	0.168	0.172	0.178	0.177	0.182

*(b) Cutoff at the short leg in AP direction*									
Normal gait/LLD (cm)									
Variables	0.0	0.5	1.0	1.5	2.0	2.5	3.0	3.5	4.0
Sensitivity	0.608	0.556	0.835	0.663	0.500	0.558	0.497	0.445	0.448
1-specificity	0.727	0.163	0.665	0.224	0.278	0.384	0.447	0.221	0.333
Cutoff	0.161	0.148	0.142	0.161	0.161	0.166	0.165	0.168	0.171

*(c) Cutoff at the long leg in ML direction*									
Normal gait/LLD (cm)									
Variables	0.0	0.5	1.0	1.5	2.0	2.5	3.0	3.5	4.0
Sensitivity	0.500	0.608	0.498	0.781	0.608	0.500	0.779	0.946	0.611
1-specificity	0.331	0.495	0.165	0.323	0.442	0.276	0.500	0.500	0.389
Cutoff	0.080	0.069	0.071	0.080	0.080	0.083	0.094	0.095	0.091

*(d) Cutoff at the short leg in ML direction*									
Normal gait/LLD (cm)									
Variables	0.0	0.5	1.0	1.5	2.0	2.5	3.0	3.5	4.0
Sensitivity	0.445	0.278	0.889	0.889	0.893	0.889	0.778	0.777	0.666
1-specificity	0.444	0.167	0.775	0.889	0.792	0.778	0.833	0.889	0.611
Cutoff	0.083	0.076	0.103	0.105	0.107	0.105	0.106	0.103	0.097

## Data Availability

The data used to support the findings of this study are available from the corresponding author upon request.
